# Monitoring livestock pregnancy loss

**DOI:** 10.7554/eLife.98828

**Published:** 2024-05-15

**Authors:** Clara Akpan

**Affiliations:** 1 Department of Veterinary Medicine, Michael Okpara University of Agriculture Umuahia Nigeria

**Keywords:** livestock reproduction, infectious diseases, disease surveillance, agriculture, Other

## Abstract

Systematically tracking and analysing reproductive loss in livestock helps with efforts to safeguard the health and productivity of food animals by identifying causes and high-risk areas.

**Related research article** Lankester F, Kibona T, Allan KJ, de Glanville WA, Buza JJ, Katzer F, Halliday JEB, Mmbaga BT, Wheelhouse N, Innes EA, Thomas KM, Nyasebwa OM, Swai ES, Claxton JR, Cleaveland S. 2024. The value of livestock abortion surveillance in Tanzania: identifying disease priorities and informing interventions. *eLife* **13**:RP95296. doi: 10.7554/eLife.95296.

Raising healthy and disease-free livestock is globally important for ensuring food and economic security as well as public health. Information about the pathogens causing livestock diseases across Africa is lacking, which makes it difficult to design strategies to prevent and control such diseases across the continent. This difficulty – combined with heat stress due to extreme temperatures and humidity – reduces livestock productivity, such as growth or milk production (Janssens et al., 2020), and increases the likelihood of livestock diseases being transmitted to humans.

Livestock abortion – where a pregnancy ends early and the foetus is expelled – is distressing for both animals and farmers, and contributes to reduced productivity and profitability of livestock projects (Keshavarzi et al., 2020). Although there are multiple potential causes of abortion, several pathogens have been associated with it globally. Therefore, collecting and analyzing data on abortion rates and their timing and associated factors could help authorities detect deviations from baseline levels that signal infections or environmental stressors that warrant further investigation (Norzin et al., 2023). This would serve as a resource for prioritizing disease control strategies (Gachohi et al., 2024), allowing policymakers to allocate resources strategically, minimizing the economic burden on farmers and the broader agricultural industry.

Due to poor disease monitoring and lack of infrastructure in Africa, little is known about the causes and impacts of livestock abortions (Dórea and Vial, 2016). Data on livestock diseases in the region rarely include information on abortion cases (Thomas et al., 2022), making it difficult to launch interventions where they are most needed. Now, in eLife, Sarah Cleaveland (University of Glasgow) and colleagues from various institutes in Tanzania, the United Kingdom and New Zealand – including Felix Lankester (Washington State University) as first author – report results from a surveillance study in northern Tanzania that aimed to identify abortion-causing pathogens and their impact on animals raised for food (Lankester et al., 2024).

The research was conducted through collaboration with the Ministry of Livestock and Fisheries, and local government authorities across Tanzania. Farmers that engage in various agricultural practices – including raising livestock alone or combined with crop cultivation or sustainable farming methods (Bodenham et al., 2021) – were encouraged to report abortion cases to livestock field officers, who then reported to the researchers. If an abortion was reported within 72 hours of it occurring, appropriate samples were collected from the females (blood, milk and vaginal swab) and the aborted foetuses. Additionally, a questionnaire was used to gather history of the livestock management, and laboratory analysis was used to test for a range of microorganisms.

A total of 215 abortion cases in cattle, sheep and goats were investigated, revealing that abortions occurred more during the dry season and in exotic and cross-bred animals rather than local livestock breeds. In 19.5% of cases, abortion was attributed to identifiable pathogens, including some that cause mild to severe illness in humans (such as *Brucella spp, Coxiella burnetii, Toxoplasma gondii* and *Rift Valley fever virus*), as well as pathogens not transmissible to humans (*Neospora spp* and *Pertivirus*). The study also identified valuable information for designing future studies. Vaginal swabs from aborting animals proved more sensitive for detecting causative agents than swabs from foetuses and the placenta. Furthermore, the longer the delay between abortion and analysis of samples, the less likely the causative agent was to be identified.

The findings suggest that surveillance of livestock abortion can be used to track important disease-causing agents responsible for reproductive loss that are not easily identified through other forms of livestock disease surveillance. This valuable information also allows monitoring of diseases that can be transmitted to humans. Additionally, the observation that more abortions occurred in non-indigenous livestock than local breeds could be used to guide herd improvement programs, for example by introducing more local livestock.

One limitation of the work of Lankester et al. is that only ten different microorganisms were tested for. In the future, expanding this number may identify more causative agents. Furthermore, increasing the number of people involved in investigation and providing suitable transport for field officers could ensure abortion cases are reported and investigated more promptly (Nansikombi et al., 2023). With the knowledge provided by Lankester et al., establishing an effective reporting and investigation system could help to design disease control measures that would be implementable even in remote rural areas.
